# Whole-Genome Shotgun Sequence of *Halomonas* sp. Strain SBS 10, Isolated from a Hypersaline Lake in India

**DOI:** 10.1128/MRA.01270-19

**Published:** 2020-01-02

**Authors:** Bijayendra Kushwaha, Guru Prasad Sharma, Anshul Sharma, Prem Shankar, Anjali Geethadevi, Nivedita Sharma, Manish Kumar Sharma, Indrani Jadhav, Deepak Parashar, Kapilesh Jadhav

**Affiliations:** a School of Life Sciences, Jaipur National University, Jaipur, India; b Blood Research Institute, Milwaukee, Wisconsin, USA; c Department of Anatomy, All India Institute of Medical Sciences, New Delhi, New Delhi, India; d Department of Laboratory Medicine, All India Institute of Medical Sciences, New Delhi, New Delhi, India; e Department of Obstetrics and Gynecology, Medical College of Wisconsin, Milwaukee, Wisconsin, USA; f National Institute of Malaria Research, Dwarka, New Delhi, India; g Department of Biotechnology, IP College, Bulandshahr, Uttar Pradesh, India; h School of Engineering and Technology, Jaipur National University, Jaipur, India; Portland State University

## Abstract

The whole-genome shotgun sequence of a moderately halophilic bacterium, *Halomonas* sp. strain SBS 10, was assembled and studied. The assembled genome size was 1.5 Mb, with a G+C content of 63.6%. The genome sequence of this *Halomonas* sp. SBS 10 isolate will be valuable in understanding gene clusters and functions involved in the adaptability of this bacterium to hypersaline conditions.

## ANNOUNCEMENT

Hypersaline environments harbor large numbers of halotolerant and moderately and extremely halophilic species that are exposed to severe stresses such as high osmolarity and low water activity (1, 2). Along with several other bacterial species, species of the genus *Halomonas* are widely distributed throughout hypersaline environments. Some of them have been recognized for their potential use in the field of biotechnology (3, 4). Analysis of their genomes will be helpful in understanding their wide genetic diversity and the genes involved in their adaptation to these stringent environmental conditions (5, 6). In our study, we isolated a moderately halophilic strain of *Halomonas* from a hypersaline lake (Sambhar Salt Lake) located in North-Western India and characterized it using whole-genome shotgun (WGS) sequence analysis.

The bacterium described here was isolated from the saltern brines of the Sambhar Salt Lake. The isolation of strain SBS 10 was carried out by streaking a sample on tryptic soy agar (TSA) supplemented with 10% NaCl. Streaking was done three times in repetition, resulting in a single colony at 37°C. The 16S rRNA gene was amplified from the genomic DNA, as described by Embley (7), with universal bacterial primers 27 F (59-AGAGTTTGATCCTGGCTCAG) and 1492 R (59-GGTTACCTTGTTACGACTT) and was aligned with those related to *Halomonas* species. The phylogenetic tree was constructed using the maximum likelihood (ML) and maximum parsimony (MP) methods available in the MEGA v. 7.0 software package (8). Phylogenetic analysis based on a 16S rRNA gene sequence (1,452 bp, GenBank accession number KT796562) comparison with the 10 closest species indicated that strain SBS 10 is clustered within the branch consisting of the species of Halomonas belonging to the Gammaproteobacteria. The isolate was found to be a member of the genus *Halomonas*, with the closest neighbor deemed to be the type strain Halomonas gudaonensis CGMCC 1.6133 (98% similarity) (9, 10).

Genomic DNA was extracted from freshly cultured tryptic soy broth (TSB) medium containing 10% NaCl using a GenElute bacterial genomic DNA kit (Sigma-Aldrich, UK), following the protocol for Gram-negative bacteria. The sequencing library was prepared using the RAD004 rapid sequencing kit (Oxford Nanopore Technologies, UK) per the instruction manual. The WGS was performed by Nanopore technology (Oxford, UK) using the MinION sequencer. In sum, a total of 71.2 Mb (316-fold coverage) was generated by the sequencing run. The quality of the 530,458 read pairs generated was assessed using FastQC v0.11.5 (https://www.bioinformatics.babraham.ac.uk/projects/fastqc/). The resulting ultralong reads were assembled using the NCBI Prokaryotic Genome Annotation Pipeline (PGAP), using SPAdes v. 3.10.1 (11). Annotation of the assembled contigs using PGAP and the GeneMarkS+ annotation method revealed 146 subsystems, 3,847 coding sequences, and 19 RNAs. For assembly and annotation using PGAP, default settings were used. Sequencing and assembly metrics for the strain are given in Table 1.

**TABLE 1:** *Halomonas* sp. SBS 10 sequencing and assembly metrics

Genome feature	Value
Sequence length (bp)	1,533,947
No. of contigs	31
Avg genome coverage (fold)	316
G+C content (%)	63.6
Shortest contig length (bp)	1,219
Median sequence contig length (bp)	43,086
Mean sequence contig length (bp)	49,482.2
Longest contig length (bp)	110,994
*N*_50_ (bp)	49,763
*L*_50_	12

### Data availability.

The genome sequence and associated data for *Halomonas* sp. SBS 10 were deposited under GenBank accession number RXHI00000000, BioProject accession number PRJNA479678, SRA accession number SRP224914, and BioSample accession number SAMN09601649.

## References

[1] Pontefract A, Zhu TF, Walker VK, Hepburn H, Lui C, Zuber MT, Ruvkun G, Carr CE 2017 Microbial diversity in a hypersaline sulfate lake: a terrestrial analog of ancient Mars. Front Microbiol 8:1819. doi:10.3389/fmicb.2017.01819. 29018418 PMC5623196

[2] Jadhav K, Kushwah B, Jadhav I 2018 Insight into compatible solutes from halophiles: exploring significant applications in biotechnology, p 291–307. *In* Singh J, Sharma D, Kumar G, Sharma NR (ed), Microbial bioprospecting for sustainable development, 1st ed Springer Nature, Singapore. doi:10.1007/978-981-13-0053-0_16.

[3] Margesin R, Schinner F 2001 Potential of halotolerant and halophilic microorganisms for biotechnology. Extremophiles 5:73–83. doi:10.1007/s007920100184. 11354458

[4] Ventosa A, Nieto JJ 1995 Biotechnological applications and potentialities of halophilic microorganisms. World J Microbiol Biotechnol 11:85–94. doi:10.1007/BF00339138. 24414413

[5] Megaw J, Gilmore BF 2018 Draft genome sequence of *Halomonas* sp. CSM-2, a moderately halophilic bacterium isolated from a Triassic salt mine. Microbiol Resour Announc 7:e00836-18. doi:10.1128/MRA.00836-18. 30533871 PMC6211355

[6] Kushwaha B, Jadhav I, Verma HN, Geethadevi A, Parashar D, Jadhav K 2019 Betaine accumulation suppresses the *de-novo* synthesis of ectoine at a low osmotic concentration in *Halomonas* sp. SBS 10, a bacterium with broad salinity tolerance. Mol Biol Rep 46:4779–4786. doi:10.1007/s11033-019-04924-2. 31230183

[7] Embley TM 1991 The linear PCR reaction: a simple and robust method for sequencing amplified rRNA genes. Lett Appl Microbiol 13:171–174. doi:10.1111/j.1472-765x.1991.tb00600.x. 1370053

[8] Kumar S, Stecher G, Tamura K 2016 MEGA7: Molecular Evolutionary Genetics Analysis version 7.0 for bigger datasets. Mol Biol Evol 33:1870–1874. doi:10.1093/molbev/msw054. 27004904 PMC8210823

[9] Wang Y, Cai H, Yu S, Wang Z, Liu J, Wu X 2007 *Halomonas gudaonensis* sp. nov., isolated from a saline soil contaminated by crude oil. Int J Syst Evol Microbiol 57:911–915. doi:10.1099/ijs.0.64826-0. 17473232

[10] Jadhav K, Jadhav I 2017 Sulfur oxidation by *Achromobacter xylosoxidans* strain wsp05 reveals ecological widening over which thiotrophs are distributed. World J Microbiol Biotechnol 30:192. doi:10.1007/s11274-017-2359-6. 28975472

[11] Tatusova T, DiCuccio M, Badretdin A, Chetvernin V, Nawrocki EP, Zaslavsky L, Lomsadze A, Pruitt KD, Borodovsky M, Ostell J 2016 NCBI Prokaryotic Genome Annotation Pipeline. Nucleic Acids Res 44:6614–6624. doi:10.1093/nar/gkw569. 27342282 PMC5001611

